# Biomedical applications of silk and its role for intervertebral disc repair

**DOI:** 10.1002/jsp2.1225

**Published:** 2022-10-06

**Authors:** Andreas S. Croft, Eugenia Spessot, Promita Bhattacharjee, Yuejiao Yang, Antonella Motta, Michael Wöltje, Benjamin Gantenbein

**Affiliations:** ^1^ Tissue Engineering for Orthopaedic & Mechanobiology, Bone & Joint Program, Department for BioMedical Research (DBMR), Medical Faculty University of Bern Bern Switzerland; ^2^ Department of Industrial Engineering and BIOtech Research Center University of Trento Trento Italy; ^3^ European Institute of Excellence on Tissue Engineering and Regenerative Medicine Unit Trento Italy; ^4^ Department of Chemical Sciences SSPC the Science Foundation Ireland Research Centre for Pharmaceuticals, Bernal Institute, University of Limerick Limerick Ireland; ^5^ INSTM, Trento Research Unit, Interuniversity Consortium for Science and Technology of Materials Trento Italy; ^6^ Institute of Textile Machinery and High Performance Material Technology Dresden Germany; ^7^ Department of Orthopaedic Surgery & Traumatology, Inselspital Bern University Hospital, Medical Faculty, University of Bern Bern Switzerland

**Keywords:** *Bombyx mori*, degeneration, functionalization, intervertebral disc, low back pain, regeneration, repair, silk, stem cells

## Abstract

Intervertebral disc (IVD) degeneration (IDD) is the main contributor to chronic low back pain. To date, the present therapies mainly focus on treating the symptoms caused by IDD rather than addressing the problem itself. For this reason, researchers have searched for a suitable biomaterial to repair and/or regenerate the IVD. A promising candidate to fill this gap is silk, which has already been used as a biomaterial for many years. Therefore, this review aims first to elaborate on the different origins from which silk is harvested, the individual composition, and the characteristics of each silk type. Another goal is to enlighten why silk is so suitable as a biomaterial, discuss its functionalization, and how it could be used for tissue engineering purposes. The second part of this review aims to provide an overview of preclinical studies using silk‐based biomaterials to repair the inner region of the IVD, the nucleus pulposus (NP), and the IVD's outer area, the annulus fibrosus (AF). Since the NP and the AF differ fundamentally in their structure, different therapeutic approaches are required. Consequently, silk‐containing hydrogels have been used mainly to repair the NP, and silk‐based scaffolds have been used for the AF. Although most preclinical studies have shown promising results in IVD‐related repair and regeneration, their clinical transition is yet to come.

## Graphical abstract

For years, researchers have been seeking a suitable biomaterial to repair and/or regenerate the intervertebral disc (IVD) and a promising candidate to fill this gap could be the application of silk. Therefore, this review aims to elaborate on the different origins from which silk is harvested, the characteristics of each silk type and how it can be used for tissue engineering purposes. Moreover, the review provides an overview of preclinical studies that have used silk‐based biomaterials to repair the inner region of the IVD, the nucleus pulposus, as well as the IVD's outer region, the annulus fibrosus.

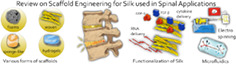

**Abbreviations**
ACAN: aggrecan
AF: annulus fibrosus
ASmiR‐214: anti‐sense miR‐214
BMP: bone morphogenetic protein
*Bombyx mori*
: *B. mori*
CAD: computer‐aided design
COL1: collagen type I
COL2: collagen type II
CS: chondroitin sulfate
ECM: extracellular matrix
GAG: glycosaminoglycan
GDF‐6: growth and differentiation factor 6
GF: growth factor
GlcNAc: N‐acetyl‐glucosamine
GMO: genetically modified organism
HA: hyaluronic acid
IDD: intervertebral disc degeneration
IVD: intervertebral disc
LBP: low back pain
MaSp: major ampullate spidroin
miRNA: microRNA
MSC: mesenchymal stromal cell
NP: nucleus pulposus
PLGA: poly(lactic acid‐co‐glycolic acid)
PTH: parathyroid hormone
RGD: arginine‐glycine‐aspartic acid
SELP: silk‐elastin‐like protein
SF: silk fibroin
SOX9: SRY‐box transcription factor 9
SS: silk sericin
TGF‐β: transforming growth factor β
VEGF: vascular endothelial growth factor

## 1 INTRODUCTION

### 1.1 The burden of low back pain and intervertebral disc degeneration

Every year, 266 million people worldwide report suffering from low back pain (LBP), with the highest incidence in Europe (5.7%).^1^ The reasons for LBP are versatile. However, the main contributor to chronic LBP is intervertebral disc (IVD) degeneration (IDD).^2^ Compared to other organs of the musculoskeletal apparatus, the onset of IDD often starts in early adolescence and then progressively aggravates with age.^3^, ^4^, ^5^ The beginning of IDD is thought to be multifactorial, and many risk factors such as excessive mechanical stress,^6^ genetics,^7^ trauma,^8^ and nutritional disorders within the IVD^9^ can set the ball rolling responsible for the progression of IDD. And once the vicious circle of IDD is entered, it is of great challenge to escape it and reverse the process.^10^ IDD usually starts in the IVD's inner region, known as the nucleus pulposus (NP).^11^ The NP acts as the IVD's central pressure and weight absorber, which it is capable of due to its highly hydrated nature and abundance of collagen type II (COL2), elastin fibers and proteoglycans like aggrecan (ACAN).^12^, ^13^ During IDD, biochemical and cellular changes occur that promote catabolic turnover.^14^ Hence, the NP's osmotic balance gets disturbed and consequently dehydrates.^11^ Decreased hydration of the NP causes a shift of the compressive load from the NP to its surrounding tissue, the annulus fibrosus (AF), which is comprised of multiple concentric ring‐like layers (lamellae) that are rich in collagen type I (COL1).^15^, ^16^ However, as the structure of the AF is preferentially made to resist tensile forces and less compressional forces, it becomes stiffer and weaker and aggravates the IVD's degeneration process overall.^15^ As a result, the IVD can bulge and cause disc herniation with associated discogenic pain.^17^

To date, the clinical management of IDD has proven to be suboptimal in many cases since the current therapy methods primarily target the symptoms of IDD, mainly pain, and not the pathophysiology itself.^18^ This issue can be largely traced back to a lack of available treatment options that would encourage the repair or regeneration of the IVD.^19^ However, as an IVD is characterized by a low cell density, low turnover, avascularity, and poor nutritional supply, it poses a considerable challenge to researchers attempting to repair it.^20^ Nevertheless, novel biomaterial‐based therapies for the treatment of IDD have attracted considerable attention in recent years.^21^ Biomaterial‐based therapies have the great advantage of preserving the IVD's structure while either already containing cells that drive the regeneration and repair or stimulating the regenerative potential of the remaining cells in the tissue.^21^

One biomaterial that has historically been used time and time again for biomedical applications is silk.^22^ Moreover, in orthopedics and especially in IVD‐related research, silk was often used to support the IVD's repair or regeneration.^23^ Therefore, this review aims to elaborate on the sources, types and properties of silk, how it has been used as a biomaterial and mainly, how the introduction of silk into IVD‐related research has been implemented to repair the damage caused by IDD and to counteract further degeneration of the IVD.

### 1.2 Silk—properties and the various species from which it can be harvested

**FIGURE 1:**
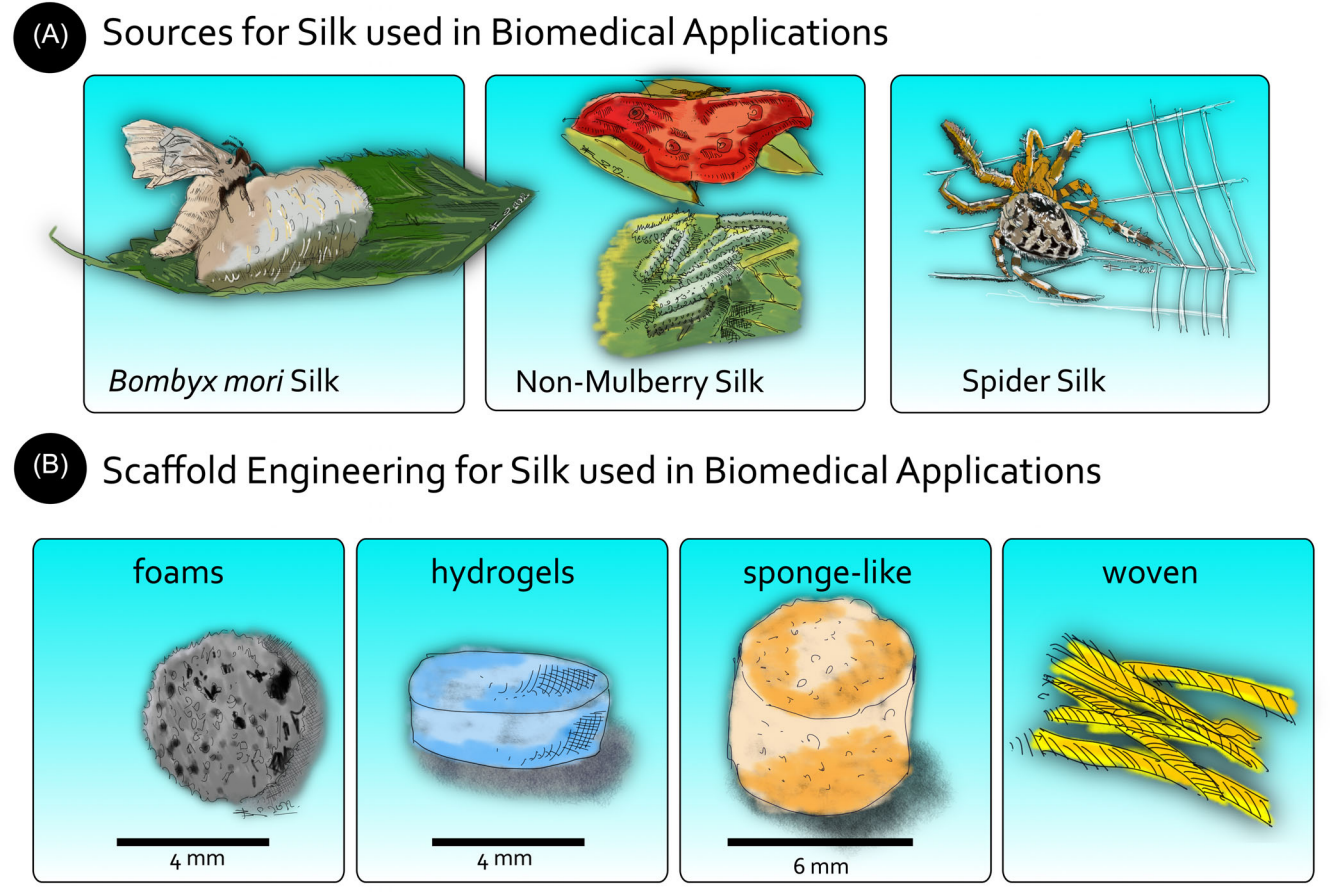
A schematic of silk origins and how it is applied for biomedical applications. (A) Sources of arthropod‐derived silk. (B) Various forms of silk scaffolds for various biomedical applications: silk foams (redrawn, based on Hardy et al.^135^), silk hydrogels (redrawn, based on Singh et al.^131^), sponge‐like silk (redrawn, based on Yu et al.^132^) and woven silk (redrawn, based on Hofmann et al.^250^)

Many insects and arachnids produce silk biopolymers as a protective shield during their life, such as the silkworms (Insecta: Lepidoptera: Bombycidae),^24^ spiders (Chelicerata: Arachnida),^25^ mites (Chelicerata: Arachnida: Acari: Tetranychidae),^26^ and wasps (Insecta: Hymenoptera).^27^ The most popular type of silk, mulberry silk, accounts for most silks produced globally (about 95%) (Figure 1).^28^ Other commercially essential types of silk are classified as non‐mulberry silk since they do not feed on mulberry plant leaves. The prominent representatives of this group are Eri silk, Tasar (Tussar) silk and Muga silk (all Insecta: Lepidoptera: Saturniidae) (Figure 1).^29^ On the other hand, there is spider silk with its outstanding mechanical properties^30^; however, its commercialization is limited by the high‐cost production and the difficulty in obtaining more significant amounts with the exception of some novel gene technology approaches.^22^, ^31^ Silks from silkworms and spiders have been widely studied for their use in tissue engineering and regenerative medicine.^32^, ^33^, ^34^ Depending on the source, the biological and physicochemical properties change due to the different structural compositions.^35^, ^36^, ^37^

#### 1.2.1 Mulberry silk

Commercially available mulberry silk is produced from a single species, i.e., *Bombyx mori* Linnaeus, 1758 (Figure 1). Mulberry silkworms are entirely domesticated, and they do not occur naturally.^38^ The silk protein is secreted from the silk glands of the mature fifth instar larva.^39^ Their cocoon is formed by a structural protein core, that is, silk fibroin (SF), surrounded by a water‐soluble coating named silk sericin (SS).^40^ SF constitutes the significant portion of the cocoon and is the core silk protein. It is the same protein reeled from the cocoons into threads to be woven into cloth that forms a significant source of income for the sericulture industry.^41^

**FIGURE 2:**
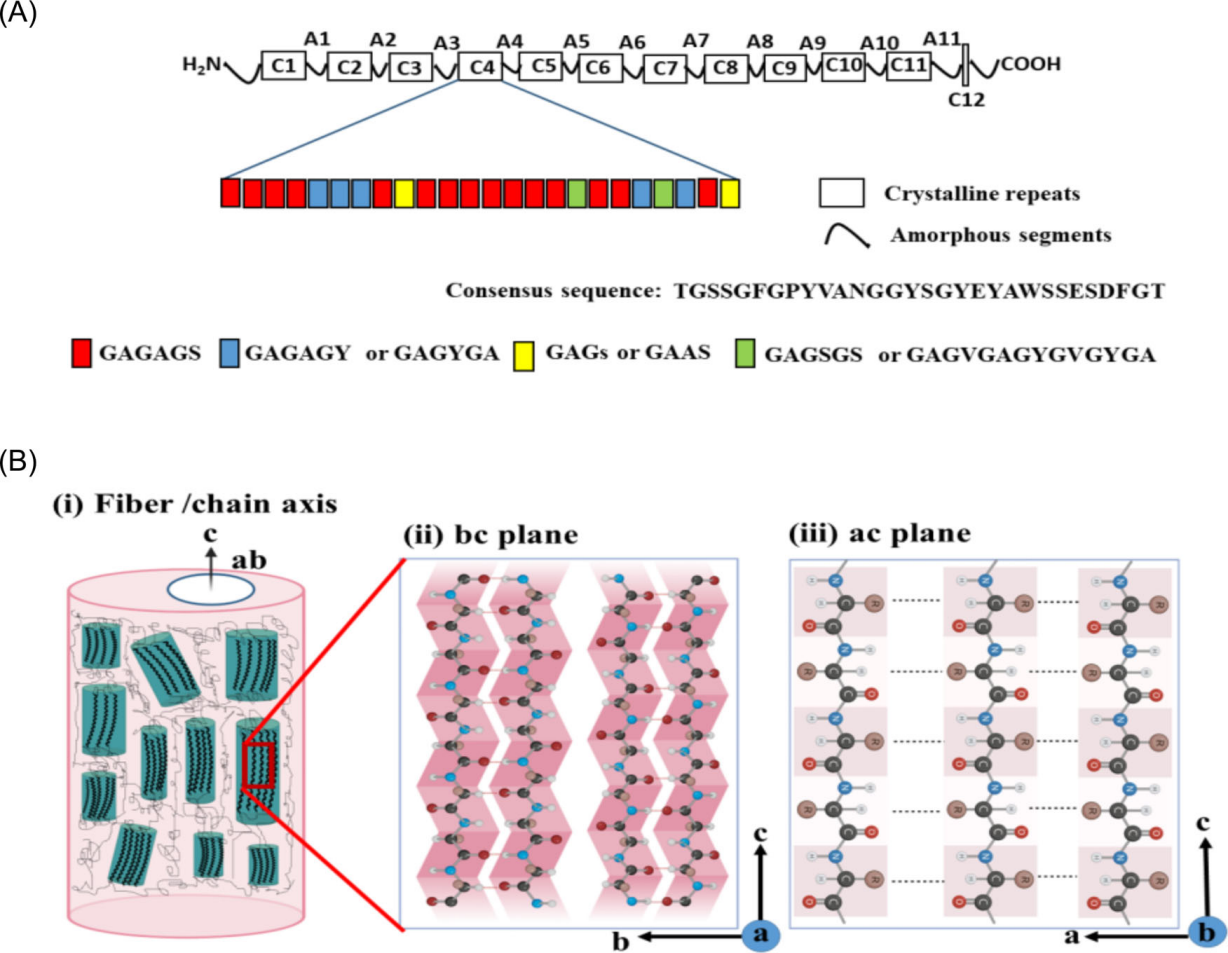
Chain configuration of silk fibroin: (A) Schematic representation of the primary structure of the silk fibroin heavy chain. Each of the 12 crystalline domains varies slightly in length and sequence, while the 11 amorphous domains are nearly identical. The amino acid sequence for one of the crystalline domains is given to highlight the repetitive nature of the protein. (B) Hierarchical structural organization in *Bombyx mori* silk fibroin. (i) Orientation of aligned beta‐sheet crystallites and amorphous regions within a native fiber. (ii) Inter‐sheet stacking within a beta crystallite, held together by van der Waals interactions between the glycine or alanine populated faces. (iii) Hydrogen bonding in the peptide chain organizes the crystalline blocks of the protein into anti‐parallel beta‐sheets

SF from *B. mori* is composed of a heavy chain (H‐chain, 360–390 kDa) and a light chain (L‐chain, 27 kDa), which are held together by a disulfide bond and a glycoprotein called P25, which is linked to both chains by noncovalent interactions in the molar ratio of 6:6:1, respectively (Figure 2).^42^, ^43^, ^44^ The primary structure of the H‐chain is a polypeptide that is mainly composed of glycine (43%–46%, G), alanine (30%, A), serine (12%, S), tyrosine (5.3%, Y) and other amino acids.^45^, ^46^, ^47^ A SF H‐chain is designed as a natural block‐copolymer with a repetitive core formed by 12 domains forming the crystalline region of SF interspersed with 11 less organized domains composed of a nonrepetitive primary sequence.^35^, ^43^ This block‐copolymer arrangement of the H‐chain guarantees the characteristic mechanical properties of SF.^48^

The other protein constituting the silk cocoon is SS, a globular protein. It is an amorphous glycoprotein that acts as a cement to keep the SF filaments together during the spinning process and comprises about 20%–30% of the cocoon's mass. Its primary structure is mainly composed of serine (28%–34%), glycine (10%–19%), aspartic acid (14%–19%), and in minor parts, other amino acids such as histidine, tyrosine, glutamic acid, threonine, and others.^49^, ^50^ The high serine content and the polar side domains of other amino acids (hydroxyl, carboxyl, or amino groups) make it highly water‐soluble. Moreover, they enable crosslinking, copolymerization, and blending.

SS obtained from *B. mori* exists mainly as random coil conformation with a molecular weight ranging between 20 and 400 kDa.^49^ A small percentage of beta‐sheets, along with beta‐turns, contributes to small crystalline domains.^51^ However, due to the principal presence of random coils, it behaves like an amorphous material, brittle in the dry state. Its properties can be improved by triggering beta‐sheet formation upon drying, mechanical stretching, moisture absorption, or chemical modifications.

The process of removing sericin from the silk fiber is named degumming. To extract and use sericin from the cocoon, different methods can be chosen: high temperature (with or without high pressure) by autoclaving, acidic solution (citric, tartaric, succinic acid), soap, and alkali solutions (sodium carbonate), highly concentrated urea or by enzymatic processes.^52^, ^53^, ^54^ With different extraction protocols, the chemical structure of sericin and its amino acid composition changes, which could impact the potential biomedical application.^54^, ^55^, ^56^ The degumming process is also fundamental for the extraction and regeneration of silk fibroin into an aqueous solution and for the structural integrity of the three subunits of the silk fibroin protein complex.^57^ Thus, it is often a crucial first step for developing silk‐based biomaterials.

In addition to SF and SS, the major silk proteins, several components of low molecular weight peptides have been identified. These silk protein components are called seroin.^58^ Seroin is distinguished from other silk proteins by high proline content, lack of cysteines, and the presence of two kinds of short amino acid repeats.^58^ It is assumed that seroin is involved in cocoon protection against predators and microbes.^59^

#### 1.2.2 Non‐mulberry silk

Representatives of non‐mulberry or “wild” silks are called tasar, eri, muga, fragaria, cricula (collectively called “Vanya silks,” coming from Sanskrit language and standing for untamed, wild, or forest‐based, Lepidoptera: Saturniidae), and shashe (Lepidoptera: Lasiocamidae) (Figure 1).^60^ The process of non‐mulberry silk production in the silkworm gland is the same as the mulberry silk. However, the spun silk displays significant characteristic differences.^61^ A prominent feature is that non‐mulberry silk has a higher cross‐section since it has reduced packing capacity due to the higher content of bulky side‐groups in the H‐chain (dibasic acids and arginine). Its stability results from higher H‐bonds in the H‐chain, which limits the dissolution. Moreover, non‐mulberry silks are more stable at high temperatures than mulberry silks and present attractive compressive strength, toughness, and elasticity.^29^, ^62^, ^63^ The amorphous domains are formed by bulky and polar side chains, responsible for maintaining the silk properties under different external treatments. Finally, non‐mulberry silk does not possess an L‐chain and the P25 glycoprotein.^64^, ^65^ The big challenge with non‐mulberry silk is the isolation and purification of SF from the cocoon. This difficulty arises from the high hydrophobic structural stability of non‐mulberry cocoons and the high amount of H‐bonds. They cannot be dissolved in lithium bromide and the other solutions used for mulberry silks. Therefore, other ionic liquids such as calcium nitrate, sodium thiocyanate, lithium thiocyanate and harsh organic solvents, such as trifluoroacetic acid, have been considered.^66^, ^67^ Due to this issue, non‐mulberry SF is generally isolated from the silk gland of the fifth instar larvae by squeezing it in a distilled water solution with anionic surfactants.^68^

A selection of non‐mulberry silks and their properties is presented in Table 1.

**TABLE 1:** Properties of various non‐mulberry fibroin and sericin

Silk type	Silkworm species	Background	Silk proteins	Molecular weight (kDa)	Breaking strain (%)	Reference
Tasar silk	*Antheraea mylitta* *Antheraea perny* *Antheraea yamamai* *Antheraea roylei*	Can be divided into two types: tropical tasar and temperate tasar. These silkworms can be bivoltine or trivoltine depending upon variety and habitat. Among non‐mulberry silkworms, *A. mylitta* has the highest silk production capacity and its cocoon is the largest.	Fibroin	Two fractions of 395 and 197	26, 27, 28, 29, 30, 31, 32, 33, 34, 35, 36, 37, 38, 39	68, 223, 224
			Sericin	Five fractions ranging from 30 to more than 200		
Muga silk	*Antheraea assama*	Muga is golden yellow colored silk and is mostly distributed in the north‐eastern region of India. The silkworms are semi‐domesticated and multivoltine.	Fibroin	Two fractions of 20 and 220	26, 27, 28, 29, 30, 31, 32, 33, 34, 35, 36, 37, 38, 39, 40, 41	223, 225, 226
			Sericin	Single fraction of 66		
Eri silk	*Philosamia ricini* (*Samia ricini*/*Cynthia ricini*)	Eri silk can regain greater amounts of moisture than mulberry silk. Its fibroin contains a many hydrophilic and positively charges amino acids. The silkworms can be domesticated, and they eat the leaves of several trees, not just mulberry leaves.	Fibroin	Two fractions of 45 and 97	24, 25, 26, 27	223, 225, 227, 228
			Sericin	Single fraction of 66		
Fagaria silk	*Attacus atlas*	*Attacus atlas* can be found in southeast Asia. Its silk has a bivoltine nature and the tensile strength of the silk yarn is greater than that of tasar and muga.	Fibroin	N/A	N/A	60, 229, 230
			Sericin	N/A		
Shashe silk	*Gonometa postica*	*Gonometa postica* is a polyphagous African insect. Its high‐quality silk has mainly been used for textiles and has only recently been implemented as a biomaterial.	Fibroin	N/A	23, 24, 25, 26, 27, 28, 29, 30, 31, 32	231, 232, 233
			Sericin	N/A		
Cricula silk	*Cricula trifenestrata*	*Cricula trifenestrata* is a silkworm from South Asian countries. The cocoons of this species are small and perforated. The silk has good biocompatible properties.	Fibroin	Single fraction of 400	12	60, 234, 235, 236
			Sericin	Single fraction of 350		

*Source*: Modified from Kundu et al.^60^

#### 1.2.3 Spider silk

Spiders produce spin silks to perform many functions, such as mating,^69^ flying,^70^ and building their webs for hunting (Figure 1).^71^ Among these, spider cobwebs are the most well‐known ones. They are composed of at least five types of silk (i.e., minor and major ampullate silks, piriform silk, flagelliform silk and aggregate silk), produced by different glands with various functional properties.^72^, ^73^, ^74^, ^75^ The most extensively characterized spider silks are from *Nephila clavipes* and *Araneus diadematus*.^22^

Spider silk provides a greater diversity of physical and mechanical properties than silkworm‐derived fibers due to multiple complex silk glands.^72^ Silk derived from the spiders' major ampullate glands is a natural hierarchically ordered material that displays a unique combination of the tensile strength (1.3 GPa), extensibility (30% elongation to fracture) and toughness (158–180 J/cm^3^). In contrast to *B. mori* silk, spider silk does not comprise any sericin.^76^, ^77^, ^78^, ^79^, ^80^ Moreover, it is biocompatible and has a very high strength‐to‐density ratio, exceeding the one of high‐performance steels and many commercial fibers.^81^ For these reasons spider major ampullate silk has always inspired researchers in the design of biomedical devices and tools with outstanding mechanical and biological properties.^32^, ^82^, ^83^

Unlike silkworms, which rely on two essential proteins—sericin and fibroin—spiders manufacture proteins (spidroins) whose composition and properties vary significantly between species.^84^ However, they consist of two nonrepetitive hydrophilic terminal domains (amino‐ and carboxy‐terminal) with a large internal repetitive hydrophobic region.^85^ Major ampullate silk is mainly composed of major ampullate spidroin (MaSp) 1 and 2 that comprise poly‐alanine and poly‐glycine‐rich domains in the repetitive region.^86^ It contains crystalline beta‐sheets formed by poly‐alanine interconnected in an amorphous matrix composed of glycine.^87^

Despite the remarkable properties, native spider silk has limited uses as it is very challenging to achieve more extensive mass production. One reason for these limitations might be the cannibalistic nature of spiders, which makes it hard to harvest silk on a large scale.^88^ A possible solution to overcome this hurdle is the production of artificial spider silk through their expression in heterologous hosts, such as bacteria.^89^ Recently, it has been proposed to use plants such as potatoes and tobacco to amplify spider silk proteins for the industry.^31^ Aside from increased scalability, protein engineering techniques allow scientists to design artificial silks with specific features that could outperform native spider silk for their use in the biomedical field, in textiles and others.^90^

## 2 SILK AS A BIOMATERIAL

Biomaterial design is a fundamental ingredient of tissue engineering. An ideal biomaterial should: (i) be biocompatible and elicit little to no host immune response, (ii) integrate physical, chemical, and biological cues to guide cells into functional tissues via cell attachment, migration, proper cell–cell interactions, cell proliferation, and differentiation, (iii) degrade at a rate favorable with new tissue formation, (iv) offer mechanical support appropriate to the level of functional tissue development, and (v) possess versatile processing options and should be easily chemically modified to suit a wide range of targeted biomedical applications.^91^

Silks represent a unique family of proteins that fulfill all these criteria of a functional biomaterial.^80^ The most studied silk type for biomaterial design is mulberry (*B. mori*) silk because it can be domesticated and regenerated in an aqueous solution. Non‐mulberry silk is not widely used in the field of biomaterials, despite the presence of the tripeptide sequence arginine‐glycine‐aspartic acid (RGD) in the primary structure, which would enhance the interaction of integrin present on the cells' surface, giving non‐mulberry silk an advantage over mulberry silk to enhanced cell adhesion and proliferation.^92^, ^93^, ^94^ As previously mentioned, the use of non‐mulberry silk, is hindered by cocoon dissolution issues, so harvesting is almost limited to direct extraction from the silk glands. Nevertheless, there are studies for its use as a potential biomaterial for different target tissues (bone,^95^ cartilage,^96^ skin,^97^ tendon,^98^ cornea^99^), for drug delivery^100^ and as tissue models.^101^, ^102^ Finally, since the spread of spider silk as a biomaterial is limited because of the difficulties in obtaining large quantities of material, there is considerable interest in the production of recombinant spider silk proteins using heterologous hosts.^88^, ^103^ To date, silk has been processed in the form of fibers,^104^ non‐woven meshes,^105^ films and coatings,^106^ porous forms,^107^ hydrogels,^108^ and bioinks^109^, ^110^ for tissue engineering purposes (Figure 1B).

### 2.1 Silk fibroin

SF from *B. mori* is an attractive biomaterial that has been used as suture material since ancient times. Meanwhile, some SF‐based products have been approved by the FDA for their use in clinics. Degummed SF yarns are used for surgical sutures and for manufacturing the knitted surgical mesh (SERI surgical scaffold™).^32^ Furthermore, a powder from freeze‐dried regenerated silk fibroin solution is used as an injectable filler (Silk Voice™) for vocal fold medialization and vocal fold insufficiency.^37^, ^111^ One reason for the excellent biocompatibility of SF may depend on the crystallinity content and the method of material processing.^112^ As an example, the thrombogenic response of regenerated SF films can be tuned by varying their beta‐sheet content and decreasing their hydrophobicity so that they adsorb more serum proteins compared to the native fibers.^113^

The regeneration of SF is fundamental for obtaining an aqueous solution that can be processed in diverse ways by controlling and triggering the self‐assembly of beta‐sheets “on‐demand” for the crystallization structure. The key features that make SF an exciting choice for tissue engineering applications are its tuneable biocompatibility,^114^ low immunogenicity,^115^ tuneable biodegradation,^116^ versatile processability,^117^ controllable and tailorable mechanical properties,^118^ and its sustainability (easy accessibility, cost‐effective and green processing).^119^

A soluble helical structure dominates the regenerated aqueous solution (Silk I‐like).^45^ When this structure is exposed to mechanical/physical and chemical treatments, the transition to Silk II occurs.^120^ Silk II is a beta‐sheet crystal‐dominated structure and is the main contributor to the SF's strength, biodegradation kinetics, biological response and insolubility in water and other solvents, such as mild acids and alkaline environments. Depending on the beta‐sheet content and the self‐assembling method, the physicochemical and biological properties of the scaffold vary.^121^

When dealing with scaffolds, it is essential that their degradation kinetics are consistent with the rate of novel tissue formation. The relevant advantage of SF is that its degradation can be controlled by tuning the crystallinity, concentration, molecular weight, the scaffold morphology, such as scaffold pore size, porosity and processing technique.^122^, ^123^, ^124^ Being a protein, SF is subjected to proteolytic digestion in vitro and in vivo by chymotrypsin, proteases, collagenases and matrix metalloproteinases.^116^ Each enzyme has a specific cleavage site, and protease XIV has been considered the most efficient for degrading silk in any material construct.^123^ Due to their long‐term functional stability, porous silk scaffolds have been used in sustainable cultures for up to 6 months.^125^

SF‐based scaffolds also display high thermal stability, depending mainly on the primary and secondary structure.^35^ Silk I and Silk II crystals melt at different temperatures, that is, 260–292°C and 286–350°C as a mean value, respectively.^126^, ^127^ Also, the processing technique and post‐treatments influence the thermal stability of SF scaffolds.^128^ Due to their thermal strength, SF‐based scaffolds can withstand different sterilization techniques, such as autoclaving, ethylene oxide, ethanol, and UV‐ and gamma irradiation, without damaging the structure.^129^ This feature is a crucial difference from many other natural polymers.

**FIGURE 3:**
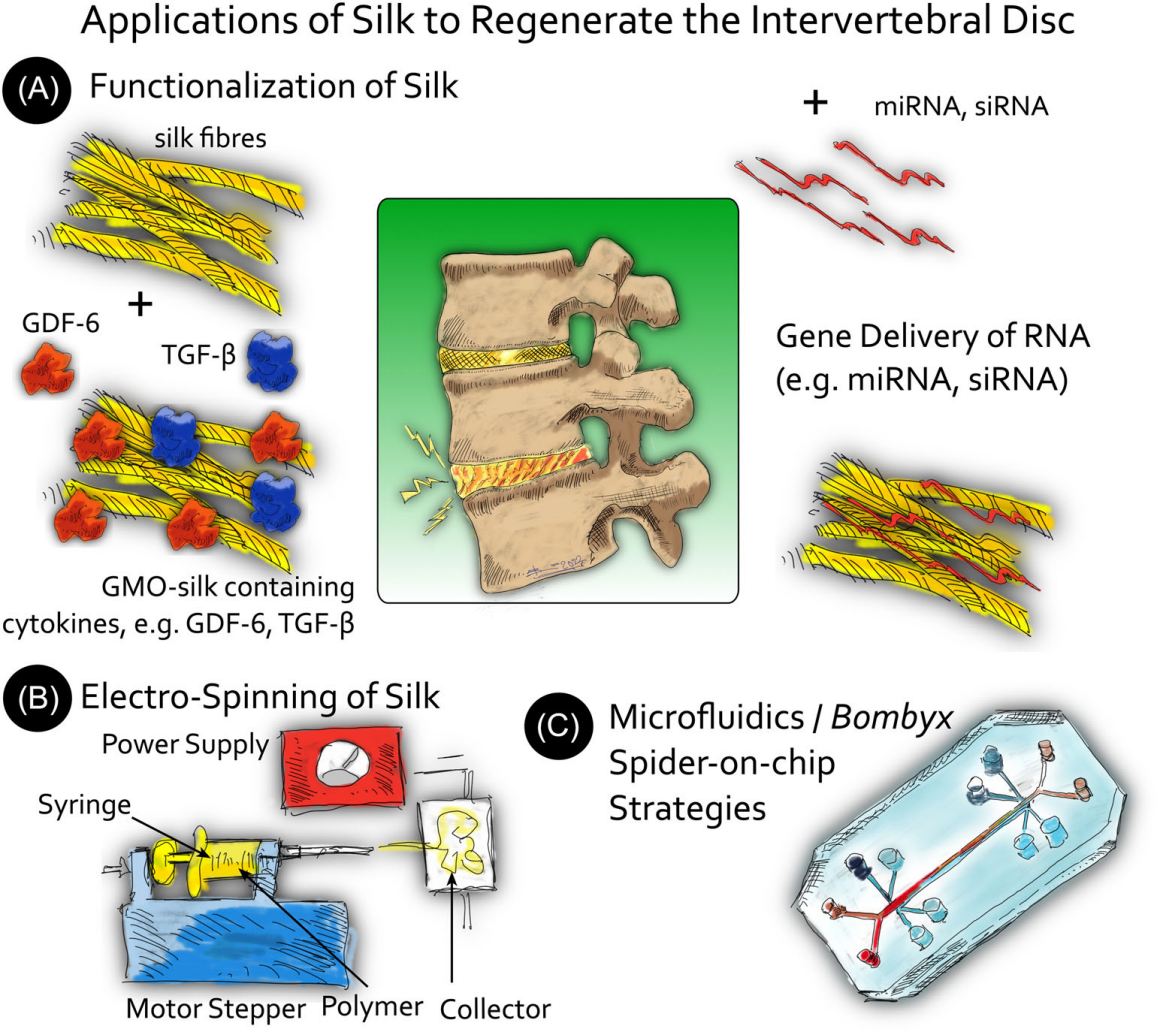
Illustration of recent advances of silk engineering for regeneration of spine applications. (A) Functionalization of silk, for example, with GMO modified *Bombyx mori* larvae overexpressing TGF‐β or GDF‐6 in silk glands based on ref or addition of RNA molecules such as miRNA, siRNA based on Frauchiger et al.^215^ (B) Process of electrospinning of silk. (C) Microfluidics of *B. mori*/spider‐on‐chip strategies

The regenerated SF solution can be processed differently to produce different scaffold morphologies such as hydrogels,^130^, ^131^, ^132^ foams^133^ and sponges,^134^, ^135^ 3D printed constructs,^136^ micro‐ and nano‐particles,^137^ electrospun membranes,^138^ and 2D films (Figures 1B and 3).^139^ This processing versatility allows it to adapt to the needs and requirements of different target tissues with diverse physicochemical and biological responses.^36^, ^113^ For example, hydrogels are highly hydrated polymer networks that can be crosslinked by other methods and permit cell seeding and encapsulation due to their capacity to retain large amounts of water. Hydrogels have been widely explored in tissue engineering because of their unique biocompatibility and biodegradability.^140^ Usually, traditional SF‐hydrogels are based on physical and chemical crosslinking (summarized in Table 2). The properties of SF hydrogels depend on the number of beta‐sheets in the material.^141^ Physical stabilization can then be achieved using shear stresses,^142^ an electric field^143^ and ultrasound^144^ or varying the temperature^145^ or pH^146^ to allow the conformational change from Silk I to Silk II. Besides physical crosslinking, however, chemical crosslinking can also be involved to improve the stability and the mechanical properties thanks to the abundance of functional groups on SF chains (i.e., tyrosine, lysine).^130^

**TABLE 2:** Types of crosslinking used for silk fibroin hydrogels

Crosslinking type	Methods	Main interactions
Physical crosslinking	Self‐assembly Ultrasonication Shear stresses Electric field application Temperature changes pH variations Organic solvents (methanol, ethanol) Surfactants (sodium lautoyl sarcosinate, sodium lauryl sulfate, poloxamer)	Noncovalent bonds (hydrogen bonding, hydrophobic interaction, electrostatic interaction, ionic interaction).^128^, ^237^, ^238^, ^239^, ^240^, ^241^, ^242^
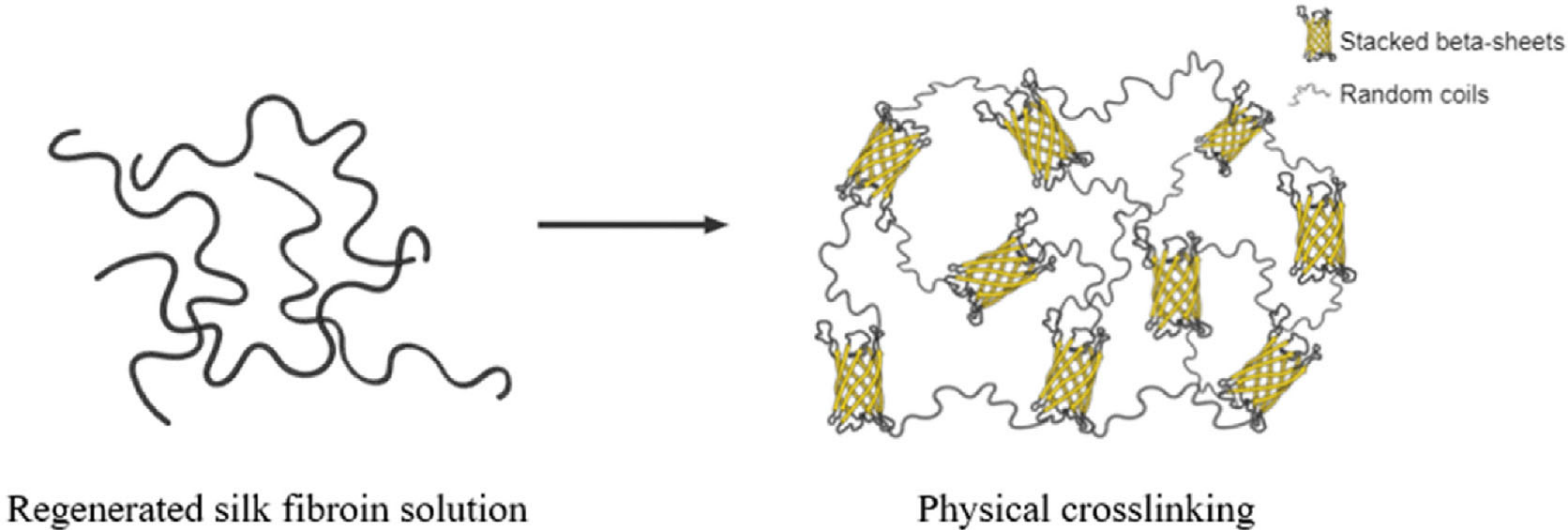		
Chemical crosslinking	Photopolymerization (UV or visible light) Irradiation (gamma‐rays) Chemical crosslinking agent (carbodiimide, genipin, glutaraldehyde) Enzyme crosslinking (horseradish peroxidase, glutamine transferase, carbonic anhydrase, alcohol oxidase, tyrosinase, laccase)	Formation of covalent bonds via enzymes, chemical agents or others.^150^, ^152^, ^243^, ^244^, ^245^, ^246^, ^247^
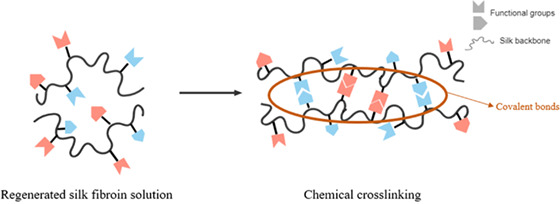		

*Note*: Sketches created in the Mind the Graph platform (www.mindthegraph.com).

Sponges and foams are interconnected porous structures whose properties can be controlled by the processing method. SF sponges can be produced by salt leaching,^147^ freeze drying,^134^ or gas foaming.^133^ They have been widely used for orthopedic applications and soft tissue engineering due to their macroporous structure, which can be adjusted for tissue regeneration and vascularization.^148^ Sponges and foams can also be used in combination with 3D printed synthetic polymer structures to promote the bioactivity of the construct. For example, in the past, SF was combined with a 3D printed polycaprolactone structure to fabricate an “entrapped in cage” scaffold for meniscus tissue engineering.^149^ The presence of silk enhanced the mechanical properties in the wet state thanks to its swelling properties and favored cell adhesion, proliferation, and metabolic activity in vitro and neovascularization in vivo.

Due to its different chemical structure, the biocompatibility of SF can be enhanced by chemical modifications of the amino acid side chains to graft bioactive molecules (peptides, growth factors [GFs]). This process includes coupling reactions (i.e., carbodiimide chemistry,^150^, ^151^ diazonium coupling^152^ or cyanuric chloride activated^153^), which can facilitate the addition of another polymer chain,^154^ oligosaccharides^155^ or specific peptide chains.^156^ A former study showed that biocompatibility was increased by the covalent addition of RGD and parathyroid hormone (PTH).^157^ The scaffold biocompatibility can also be improved by blending SF with other materials, i.e. with calcium phosphates or specific inorganic components to enhance osteogenic properties.^158^ Furthermore, silk fibroin scaffolds have also been shown to promote differentiation of mesenchymal stromal cells (MSC) with extracellular matrix (ECM) secretion and mineralization, making them an optimal candidate for orthopaedical tissue regeneration.^159^ Moreover, the incorporation of specific GFs (i.e., bone morphogenetic protein 2 [BMP‐2],^160^ BMP‐7,^161^ vascular endothelial growth factor [VEGF])^162^ further increased the osteogenic and angiogenic potential of SF scaffolds.

### 2.2 Silk sericin

SS is a natural polymer protein material produced by the silkworm *B. mori* that covers and holds the silk fibroin filaments together. However, SS is usually considered a side‐product of the cocoon during the degumming process, becoming an unutilized waste product. Researchers started to extract it as biomaterial due to its natural origin, availability, and interesting biological properties. SS has been recognized as the immunogenic element of the silk filament for years, which has fuelled research into the purification of SF and its regeneration. However, researchers demonstrated that the immunogenicity of the silk fiber is mainly due to the combination of SS with SF, although the mechanism responsible for initiating the immune response is not yet fully understood.^163^

SS has been used in cosmetics for years due to its properties such as antioxidant,^164^ moisturizing,^165^ UV‐protective potential,^166^ and oxygen permeability.^167^ For tissue engineering approaches, SS‐biomaterials have been synthesized in various forms, such as hydrogels,^168^ sponges,^169^ films,^170^ and inks.^171^ Furthermore, with the development of SS‐based 3D scaffolds and films, its biological effects could be investigated. SS showed an increase in the migration, proliferation and production of COL1 in skin cells.^172^ Additionally, it favors the growth of keratinocytes and fibroblasts, which makes it a potential candidate for epithelial tissue repair and wound dressings.^173^ Moreover, SS can favor the nucleation of bone‐like hydroxyapatite, raising interest in its use in bone tissue engineering and the coating of titanium surfaces.^174^, ^175^ Finally, due to its chemical reactivity, pH sensitivity and amphiphilic structure, SS has been employed for the design of drug delivery systems and for targeting purposes.^174^

Despite the interesting biological properties, the widespread use of SS‐based scaffolding materials is limited because it is characterized by fast degradation rates and weak mechanical properties. However, thanks to the presence of hydroxyl, carboxyl and amino groups present in the polar side chains, it can be crosslinked,^176^ co‐polymerized^177^ and blended^178^ with other polymers to improve biomechanical features or to conjugate bioactive molecules. For example, in a previous study, SS was combined with gelatin methacrylate, and it was then used as ink for 3D printing purposes favoring the proliferation and stratification of keratinocytes.^171^

### 2.3 Functionalized silk

As mentioned above, silk as a biomaterial already has a lot of advantages in tissue engineering applications. However, when silk is fabricated into a scaffold with designed functions and in contact with the tissues, the microenvironment with which the scaffold is in contact is complicated, so the requirements of the material are more crucial. For this reason, the silk or silk‐based scaffold should be functionalized to have a controllable performance according to the final purpose. The general principle of functionalization is the use of physical or chemical methods to make silk as a delivery and sustainable controlled‐release system of some targeted molecules, in order to improve the tissue‐specific biological properties of the scaffold.

#### 2.3.1 Growth factor and cytokine functionalization on silk

GFs and cytokines are two major biological signal molecules, which regulate cellular function, and have an essential contribution to ECM synthesis.^179^ Since silk is a well‐studied material in this field, functionalized silk with GFs and cytokines has presented excellent performances in both delivering and releasing. In the past, the biological properties of the dual GFs BMP‐2 and transforming growth factor β1 (TGF‐β1) functionalized silk‐based (non‐mulberry silk fibroin, from *Antheraea mylitta*) scaffold have been studied for bone regeneration using different functional methods. Bhattacharjee et al.^180^ loaded the two GFs using the carbodiimide‐coupling reaction, while Naskar et al.^181^ loaded the same GFs by simple physical blending. Even though the architectures of these two studies were completely different, both results showed that the functionalized silk scaffolds had a sustained GF release profile, good cell adhesion, proliferation, and migration, as well as an earlier stage differentiation. In the study by Wang et al., osteochondral GFs were either encapsulated in poly(lactic acid‐co‐glycolic acid) (PLGA)‐based or mulberry SF‐based microspheres and then further incorporated in alginate or silk scaffolds to create concentration gradients.^182^ The results showed that both microsphere types were able to form concentration gradients and induced human MSCs to differentiate along the concentration gradient into an osteochondral phenotype.

#### 2.3.2 Functionalizing silk with miRNA

MicroRNAs (miRNAs) are short, noncoding RNA molecules that regulate gene expression. Importantly, due to the “small size” of these RNAs, the therapeutic miRNAs will not integrate into DNA, thus eliminating the scruples about genetic alterations (Figure 3A).^183^ Although the regulation of miRNA on skeletal tissues is widely studied, the application of miRNA to functionalize silk or silk‐based scaffolds for tissue engineering is still an emerging field. One such sparse study where miRNA‐functionalized‐silk was used for orthopedic research was conducted by James et al.^184^ Here, they developed an all‐aqueous, silk‐based device to enhance the osteoinduction of MSCs. Just by simply doping the silk fibroin solution blended with anti‐sense miR‐214 (ASmiR‐214) on the surface of the silk‐based screw, the continuous release of miRNA that inhibits the expression of osteoinductive antagonists could be detected up to 7 days. The in vitro evaluations demonstrated that the osteoblastic commitment and osseous integration were enhanced.

#### 2.3.3 Microfluidics using silk

Recently, lab‐on‐chip approaches were followed for the production of nano‐particles of silk and nano‐films (Figure 3C).^185^, ^186^ Also, bioinks for the usage in 3D printing engineering have been developed.^187^, ^188^ Jeon et al. for instance, investigated on silk‐elastin‐like protein (SELP) polymers that can be used for predicted drug release depots.^189^ Peng et al. mimicked the complex interplay of different silk glands of spiders as a “spider‐on‐chip” approach.^190^ Silk in general seems to be very advantageous for the usage of lab‐on‐chip devices compared to other materials such as ceramics and polymers.^191^, ^192^ This is specifically true if advantage is taken of hydrophilic or hydrophobic yarns with high elasticity.^191^, ^193^, ^194^

## 3 SILK USED FOR INTERVERTEBRAL DISC REPAIR

Regarding the silk's biomechanical properties and its versatile biomedical applications, this biomaterial has also been considered and used for IVD research with the aim to repair damaged and/or degenerated IVDs.^23^ Anatomically spoken, there are two approaches how to repair the IVD using silk; either by targeting the NP or the AF.

### 3.1 Nucleus pulposus repair

In the past, the NP has been a popular target to repair a degenerated IVD. This is likely related to the fact that IDD has its origins in the NP, where a reduced ECM turnover accompanied by a loss of internal proteoglycans leads to the disc's dehydration.^11^ Most NP replacement biomaterials, made at least partially of silk, are hydrogels. This seems like the most obvious choice since hydrogels and the NP both share a lot of common ground. Hydrogels can absorb significant amounts of liquid, and they can be designed to have similar mechanobiological properties as the highly hydrated NP.^195^, ^196^, ^197^

One of the first approaches to use a hydrogel with incorporated silk to regenerate NP tissue was carried out by Park et al.^198^ Their approach was to encapsulate chondrocytes with a hydrogel that consisted of fibrin/hyaluronic acid (HA) only, 2% silk or a combination of both biomaterials either with 1%, 1.5%, or 2% silk. They supplemented silk to the fibrin/HA hydrogel to achieve superior mechanical strength compared to plain fibrin/HA gels. After 1 week of culture, all five groups showed a defined chondrogenic area stained with alcian blue. Furthermore, all silk groups expressed a significantly higher GAG content than the fibrin/HA only group after 1 week. However, based on the gene expression of *COL2*, *SOX9*, and *ACAN*, the 2% silk gel and the 2% hybrid were inferior to the other groups. Interesting results were also found regarding the mechanical properties of the different gels. As hypothesized, all samples treated with silk presented a significantly higher compressive modulus and yield strength than those without silk, making them a better substitute for NP tissue.

Recently, a similar approach has been carried out that also explored the influence of silk/HA‐composite hydrogel concentrations on the samples' biomechanical behavior.^199^ This study confirmed the correlation between higher silk to hyaluronic acid ratio and a higher viscoelastic modulus. Furthermore, in combination with TGF‐β3 enriched chondrogenic inductive medium, the hydrogels promoted considerable NP‐like differentiation of bone marrow‐derived MSCs after only 7 days. This differentiation was most clearly noticeable through a significantly enhanced expression of GAG and COL2 in the ECM and a significantly higher gene expression of *ACAN* and *COL2*.

Another study on silk‐based hydrogels was conducted by Hu et al.^200^ Here, they were working on an injectable hydrogel with the ultimate goal of replacing a degenerated NP. They proposed a crosslinked hydrogel composed of silk fibroin and polyurethane, which could be prepared in a liquid or semi‐liquid state at room temperature. The hydrogel showed great cytocompatible properties during a one‐week culture period using bone marrow‐derived MSCs, good radiographical visibility and a Young's modulus comparable to that of a natural NP. In a follow‐up study, the group further focused on the mechanical features of the same hydrogel as well as its in vivo biocompatibility.^201^ Confined compression and fatigue tests revealed adequate physical‐mechanical characteristics and the ability to withstand a million cycles at an axial strain of 15% and a frequency of 5 Hz. The hydrogel's biocompatibility was shown with an in vivo rabbit model, where they transplanted the dried implants into the paravertebral muscle. After 3 months, no inflammatory response was observed in the surrounding tissue, nor did the hydrogel display any apparent signs of deformation or degradation. In conclusion, the investigators state that due to the good biomechanical properties, which resemble those of a healthy NP, further animal trials and, ultimately, its clinical transition seems realistic.

The wide range of possible applications of silk fibroin for the regeneration of the NP was nicely shown by Murab et al.^202^ Not only was the silk used as a hydrogel for improved mechanical support, but within the hydrogel, silk fibroin was shaped into hollow microspheres and used as carriers for N‐acetyl‐D‐glucosamine (GlcNAc). As GlcNAc has been known to regulate the expression of TGF‐β1^203^ and for the formation of large proteoglycan aggregates,^204^ it was hypothesized that its spatiotemporal release would enhance the differentiation of human adipose‐derived stem cells towards a NP‐like phenotype. Indeed, cells cultured in hydrogels containing GlcNAc‐loaded microspheres expressed significantly more *COL2* and *ACAN* than controls lacking GlcNAc. Furthermore, the hydrogel's rheological characterization demonstrated its injectability, and cyclic compressive testing using degenerated IVDs with subsequent hydrogel injection revealed a compressive strength similar to that of a healthy IVD.

Almost all trials that aimed to regenerate/repair the NP with silk used such from the silkworm *B. mori*. In this context, only a single study has assessed how non‐mulberry silk could be used for NP regeneration.^196^ Here, composite hydrogels were formed using different ratios of silk fibroin proteins derived from *Antheraea assamensis* and *B. mori*. The aim was then to find a suitable mixture of these two silk fibroins for in situ replacement of the NP. After testing different ratios of silk fibroin blends, the investigators observed that the higher the concentration of *Antheraea assamensis* derived silk, the faster the hydrogel's gelation, but also degradation time, the higher the proliferation rate of NP cells and the greater its ability to swell as well as to withstand cyclic compression. In conclusion, they state that the hydrogel's properties can be adjusted by varying the silk fibroin proportions, making it a potential candidate for clinical translation.

Nevertheless, despite the promising results obtained with hydrogels for NP repair, they also have their limitations. Due to their viscous nature, cell migration and the exchange of nutrients and waste products into and out of the hydrogel can be hindered, and the synthesis of newly formed ECM can be impaired.^205^, ^206^ To tackle these issues, Zeng et al. created a highly porous silk fibroin scaffold with interconnected macropores, leaving enough space for NP cells to infiltrate, increase, and to deposit newly synthesized ECM.^206^ As hypothesized, NP cells infiltrated the scaffolds and proliferated well therein, as a significant increase in DNA content was found over a culture period of 3 weeks. Moreover, quantitative analysis showed that significantly more COL2 and proteoglycans were deposited on the scaffold after 3 weeks of culture, which then further improved the compressive elastic modulus of the scaffold itself.

A summary of published articles concerning the application of silk‐based biomaterials for the repair and regeneration of the NP can be found in Table 3.

**TABLE 3:** Overview of published studies where silk was used to repair/regenerate the nucleus pulposus

Silk origin	Silk structure	Study	Conclusions	References
*Bombyx mori*	Hydrogel	Assess whether a composite hydrogel made of silk‐fibrin and hyaluronic acid causes greater mechanical strength and more chondrogenesis than silk‐fibrin/hyaluronic acid alone.	Silk‐fibrin/hyaluronic acid hydrogels had improved mechanical strength and a decreased degradation rate while maintaining the chondrogenic phenotype of NP cells.	198
*Bombyx mori*	Hydrogel	Comparing the mechanical properties and chondrogenic inductive potential of hydrogels made of hyaluronic acid and different silk fibroin strains with varying weight ratios.	The higher the weight ratio of silk fibroin to hyaluronic acid, the greater the viscoelastic modulus. Moreover, the hydrogels promoted NP‐like differentiation of MSCs.	199
N/A	Hydrogel	Testing the biomechanical properties of a NP replacement hydrogel consisting of silk fibroin and polyurethane.	The hydrogel possessed adequate physical‐mechanical properties to replace the NP as a prosthetic biomaterial.	200
N/A	Hydrogel	Determine the compressive mechanic characteristics, stability and biocompatibility of a composite hydrogel made of silk fibroin and polyurethane in vivo.	The hydrogel showed great biocompatibility in vivo and no obvious signs of degradation were found after 3 months of implantation.	201
*Bombyx mori*	Cryogel	Assess the effect of a silk fibroin enriched poly (vinyl) alcohol cryogel on its hydrophilicity and how it influences the cellular attachment and proliferation of adipose‐derived MSCs.	The enrichment of silk improved the cryogels' rehydration ratio, water content, hoop stress, and compressive modulus. Moreover, cell‐hosting abilities were improved.	248
*Bombyx mori*	Hydrogel	Creating a hydrogel that resembles the NP's ECM. The hydrogel was chitosan and COL2 based and to increase the hydrophilicity and stability, gelatin and silk fibroin were added.	The hydrogel was injectable at 4°C and started gelation after 30 min at 37°C. The addition of silk increased the hydrogel's stability and durability.	249
*Bombyx mori*	Silk microspheres embedded in a silk hydrogel	Study the effect of GlcNAc loaded hollow spheres on the NP‐like differentiation of adipose‐derived MSCs, which are embedded in silk fibroin together with the spheres.	Spatiotemporally controlled release of GlcNAc enhanced the expression of COL2, ACAN and GAG. Furthermore, the hydrogel brought adequate structural support during cyclic compression.	202
*Bombyx mori* and A*ntheraea assamensis*	Hydrogel	Blending two different silk variations to design a suitable hydrogel for in situ NP replacement applications.	The gelation time and mechanical properties of the hydrogel could be tuned depending on the ratio of the silk variants. NP cells proliferated on all variants tested.	196
*Bombyx mori*	Scaffold	Determine the feasibility of porous silk fibroin scaffolds seeded with NP cells for NP regeneration.	NP cells proliferated in the scaffold and produced significant amounts of COL2 and proteoglycans. A higher cell number resulted in a greater compressive elastic modulus of the scaffold.	206

Abbreviations: ACAN, aggrecan; COL2, collagen type II; ECM, extracellular matrix; GAG, glycosaminoglycan; GlcNAc, N‐acetyl‐glucasamine; MSC, mesenchymal stromal cell; NP, nucleus pulposus; N/A, non‐available.

### 3.2 Annulus fibrosus repair

Just as hydrogels have been used almost exclusively for the repair/regeneration of the NP, only firm scaffolds can be found for the application of the AF. Early investigations on how to implement silk specifically for the repair or regeneration of the AF were done by Park et al.^207^, ^208^ In two related studies, silk scaffolds with a lamellar morphology were compared to ones with a porous, spongy structure. The aim was to find out whether an AF‐like lamellar structure would positively influence the tissue formation of porcine AF cells. Both studies concluded that the lamellar orientation of the scaffolds improved the construction of AF‐like tissue during a culture period of 2 weeks. Furthermore, compared to the porous scaffolds, the amount of GAG gradually and significantly increased in the lamellar samples, and significantly more collagen was detected towards the end of each experiment. Surprisingly, however, the porous scaffold displayed a superior elastic modulus and tensile strength after 1 day of culture but then showed comparable values after 2 weeks.

Around the same time, See et al. worked on silk scaffolds for AF regeneration. In a first attempt, cell sheets consisting of bone‐marrow‐derived MSCs were transferred onto a porous silk scaffold and wrapped around an artificial NP made of silicone.^209^ The IVD‐like assembly was then cultured for 4 weeks under static conditions. During this culture period, the cell activity remained unchanged, and the cell sheets' initial COL1‐rich ECM shifted towards a COL2‐dominant environment, resembling the ECM found in the inner AF. The same construct was then used in a follow‐up study, but this time they cultured it in a bioreactor that enabled dynamic compressional loading.^210^ Results revealed that, on the one hand, the MSCs' metabolic activity decreased significantly after 4 weeks of culture compared to the static load. On the other hand, dynamic loading improved the gene expression profile of the MSCs that were seeded onto the scaffold, as essential IVD‐related genes such as *SOX9*, *COL1*, *COL2*, *ACAN*, and *biglycan* were significantly higher expressed than in the static condition. Since then, multiple studies have attempted to mimic the AF's anatomical structure using a silk scaffold, and they all share the same conclusion: The more precisely the scaffold can replicate the human AF, the better the phenotype of the cultured cells and the closer the mechanical properties of the scaffold compared to native AF tissue.^211^, ^212^

Although the morphology of the scaffold has a crucial impact on the synthesis of AF‐like tissue, the composition and the properties of the biomaterial itself are also known to be just as ground‐breaking for successful tissue formation. The surface of silk fibroin can be functionalized by covalent conjugation of biomolecules, thereby making its features tuneable and consequently allowing a more efficient and better‐defined differentiation or retention of an AF‐like phenotype.^213^ For example, RGD functionalization of silk fibroin scaffolds has shown to enhance the expression of *ACAN* and *COL2* in AF cells compared to the nonmodified silk.^214^ Another example would be the application of genetically engineered silk that was functionalized either with TGF‐β3 or growth and differentiation factor 6 (GDF‐6) and was able to preserve the phenotype of human AF cells (Figure 3).^215^ Ideally, however, surface functionalization is combined with a suitable scaffold that tries to imitate the structure of the AF as closely as possible. The functionalization was nicely illustrated in a series of studies by Bhattacharjee et al.^216^, ^217^, ^218^ Here, silk fibroin fibers were functionalized with chondroitin sulfate (CS) and were aligned in a crisscross orientated manner to resemble the AF. Although first attempts could not reveal any notable differences between the functionalized and the plain scaffold,^216^ a refined setup using articular chondrocytes instead of nasal chondrocytes led to an upregulation of *SOX9*, *ACAN*, and *biglycan*, an improved production of GAG and collagens, an eminent increase of the metabolic activity and a significantly higher compressive strength with the CS‐treated scaffolds compared to controls.^217^ However, the full potential of their functionalized scaffold was achieved when the scaffolds were cultured in a hydrodynamic environment and a distinction was made between inner and outer AF.^218^ Previous studies had shown that the presence of CS can cause MSCs to adopt a phenotype comparable to that of inner AF cells.^219^, ^220^ Consequently, only the inner part of the scaffold was functionalized with CS. With this study design, they demonstrated that the dynamic culture conditions enhanced the metabolic activity and the production of ECM. Furthermore, due to the CS in the inner AF, a chondrogenic tissue gradient was formed, with the inner part expressing significantly more *COL2*, *ACAN*, *biglycan* and significantly less *COL1* and *elastin* than the outer part of the scaffold, thereby mimicking the properties of native AF tissue.

A relatively recent and very ambitious study on the use of anatomically‐shaped silk scaffolds for the regeneration of the AF was conducted by Costa et al.^221^ Therefore, a reverse engineering approach was followed in which a human patient was subjected to an MRI scan and then, based on the segmented morphologic scan of the L1‐L2 IVD, a 3D model of the AF's ultrastructure was printed with a bioink made of SF and elastin. Remarkably, the mechanical characteristics, including the compressive modulus and stress–strain‐curve, turned out to be very similar to those of fibrocartilage cartilage tissue found in the AF.^221^ Moreover, human adipose‐derived stem cells adhered well onto the scaffold and remained metabolically active for 21 days.

Finally, it is important to point out that silk scaffolds or scaffolds in general do not always have to be the basis for novel tissue formation and as a result aim to replace the defective or degenerative tissue itself. Still, they can be also used specifically to support the reparative process of a defective site. A corresponding example of this is provided by Frauchiger et al. using a bovine IVD damage model.^222^ Here, an AF defect was induced with a biopsy punch and the created cavity was subsequently filled with a genipin‐enhanced fibrin hydrogel, sealed with a silk fibroin scaffold and then the entire IVD was tested under different culture/loading regimes. Even though the IVDs' height could not be recovered, the silk scaffold reliably sealed the repaired site, as no herniation of the hydrogel could be detected during extensive dynamic loading.

A summary of published articles concerning the application of silk‐based biomaterials for the regeneration and repair of the AF can be found in Table 4.

**TABLE 4:** Overview of published studies where silk was used to repair/regenerate the annulus fibrosus

Silk origin	Silk structure	Study	Conclusions	References
*Bombyx mori*	Scaffold	Assess whether either lamellar or porous spongy silk scaffolds are better suited for AF tissue formation and function. Both scaffolds were seeded with porcine AF cells.	Both scaffolds showed similar mechanical properties after 2 weeks of culture. However, lamellar scaffolds enabled a significantly higher GAG and collagen production than porous scaffolds.	207
*Bombyx mori*	Scaffold	Creating a biphasic structure that mimics the IVD. The AF‐like structure was made of silk and was cultured with porcine AF cells and the NP scaffold consisted of fibrin and hyaluronic acid and was cultured with porcine chondrocytes.	The amount of GAG significantly increased with the lamellar AF scaffolds during a culture period of 4 weeks. Lamellar NP scaffolds showed significantly more collagen after 2 weeks and more GAG after 4 weeks.	208
*Bombyx mori*	Scaffold	Assess whether porous silk scaffolds with or without RGD allow AF cells to attach and promote ECM production.	AF cells attached and proliferated on the scaffold regardless of whether the silk was functionalized with RGD or not. However, RGD silk improved *ACAN* and *COL2* expression.	214
N/A	Scaffold	Integrating MSC‐sheets onto silk scaffolds that were wrapped around a disc made of silicon. The artificial IVD was cultured for 4 weeks in static conditions.	MSCs‐sheets adhered well to the silk scaffolds. During a culture period of 4 weeks, MSCs remained metabolically active and the COL2 to COL1 ratio increased over time.	209
N/A	Scaffold	Using alternating layers of silk scaffolds that were wrapped around a silicon disc to simulate and IVD‐like assembly. MSC‐sheets were put onto the scaffolds and the construct was mechanically stimulated.	Cells remained viable over a culture period of 4 weeks, however, the viability gradually decreased. Mechanical stimulation guided the MSCs to differentiate towards a phenotype that resembles the inner AF.	210
*Bombyx mori*	Scaffold	A multilayered, angle‐ply scaffold was created to mimic the anatomical structure of the AF. Porcine AF cells or human MSCs were seeded onto the scaffold.	AF cells and MSCs proliferated during 14 days of culture and produced significant amounts of ECM. The scaffolds also displayed good mechanical properties.	211
N/A	Scaffold	Creating a biomimetic multilamellar angle‐ply AF‐like scaffold made of polycaprolactone and silk fibroin fibers with ±30° alternating orientation. Leporine AF cells were seeded onto the scaffold.	The AF‐like scaffolds possessed mechanical properties similar to those of a natural AF. AF cells managed to adhere, proliferate, infiltrate into the scaffold and deposited ECM.	212
*Antheraea mylitta*	Scaffold	Creating a silk fibroin scaffold using crisscross‐oriented fibers to mimic the structure of a native AF tissue. Fibers were made with or without crosslinked CS.	Nasal chondrocytes aligned along the silk fibers and produced GAG and COL2 after 4 weeks of culture, regardless of the presence of CS.	216
*Antheraea mylitta*	Scaffold	Study the cellular response of CS functionalized silk scaffolds using articular chondrocytes.	Chondrocytes showed enhanced chondrogenic redifferentiation potential and a higher metabolic activity in the presence of CS functionalized silk.	217
*Antheraea mylitta*	Scaffold	Mimicking the inner and outer AF using crisscross‐orientated silk fibers and articular chondrocytes. The fibers of the inner AF were functionalized with CS. The scaffolds were loaded statically and dynamically.	A tissue gradient was formed that mimicked the characteristics of the inner and outer AF. Cells in the inner AF produced more GAG and expressed more *COL2* and *ACAN*, whereas more *COL1* was found in the outer AF.	218
*Bombyx mori*	Scaffold	3D printing of an anatomically‐shaped AF using a composite bioink made of silk fibroin and elastin. Adipose derived stem cells were used to test the scaffold's cytocompatibility.	The bioprinted scaffolds morphologically resembled an anatomically‐shaped AF and displayed mechanical characteristics similar to a native AF. Moreover, cells were metabolically active for 21 days.	221
*Bombyx mori*	Scaffold	Assess how well genetically engineered silk containing TGF‐β3 or GDF‐6 promotes IVD‐like differentiation of MSCs and how well it maintains the phenotype of AF cells.	MSCs expressed about 10 times more *ACAN* than *COL2*, indicating a trend towards NP‐like differentiation. AF cells were not negatively affected by the silk.	215
*Bombyx mori*	Scaffold	An AF injury was induced in bovine IVDs. To repair the IVD, the created cavity was filled with a genipin‐enhanced fibrin hydrogel and sealed with a silk scaffold. Then, the IVDs were mechanically tested.	The repair was considered as a success because no herniation occurred regardless of the loading condition. However, the IVDs' height could not be recovered.	222

Abbreviations: ACAN, aggrecan; AF, annulus fibrosus; CS, chondroitin sulfate; COL1, collagen type I; COL2, collagen type II; ECM, extracellular matrix; GAG, glycosaminoglycan; GDF‐6, growth and differentiation factor 6; IVD, intervertebral disc; MSC, mesenchymal stromal cell; NP, nucleus pulposus; RGD, arginine‐glycine‐aspartic acid; TGF‐β3, transforming growth factor β3; N/A, non‐available.

## 4 CONCLUSION

Humans have bred silkworms for centuries, and their silk has found many uses thanks to its excellent biomechanical properties. There are many different species from which silk can be harvested, and each has its characteristics, advantages, and disadvantages. However, the clear dominator on the market is SF, derived from the silkworm *B. mori*.

In the clinic nowadays, silk is mainly used for surgical sutures. However, many preclinical studies show its great potential and versatility as a tissue repair and regenerative biomaterial. Especially in the past decade, the application of silk has also found its way into the field of IVD‐related research, where mainly silk‐based hydrogels have been used for the regeneration of the NP and only firm silk‐based scaffolds have been investigated for the repair of the AF. Some of these scaffolds and hydrogels show auspicious outcomes. They thus indicate a transition into clinics in the foreseeable future, where they will hopefully present themselves as another missing puzzle piece to treat and ultimately cure patients suffering from IDD.

## AUTHOR CONTRIBUTIONS

The study conception and design were proposed by Andreas S. Croft and Benjamin Gantenbein. The literature search and data analysis were done by Andreas S. Croft, Eugenia Spessot, Promita Bhattacharjee, and Yuejiao Yang. Benjamin Gantenbein, Promita Bhattacharjee and Eugenia Spessot designed the figure arts. The initial draft of the manuscript was written by Andreas S. Croft, Eugenia Spessot, Promita Bhattacharjee, and Yuejiao Yang and was then reviewed and edited by Antonella Motta, Michael Wöltje, and Benjamin Gantenbein. Funding was provided by Benjamin Gantenbein, Antonella Motta and Michael Wöltje.

## CONFLICT OF INTEREST

The authors declare no conflicts of interest.
